# Perioperative management of patients with severe pulmonary hypertension in major orthopedic surgery: experience-based recommendations

**DOI:** 10.3205/iprs000062

**Published:** 2015-01-14

**Authors:** Hans-Jürgen Seyfarth, Jochen Gille, Armin Sablotzki, Stefan Gerlach, Michael Malcharek, Andreas Gosse, Ralf H. Gahr, Elke Czeslick

**Affiliations:** 1 Medical Clinic and Polyclinic I, Department of Pneumology, Universitätsklinikum Leipzig AöR, Leipzig, Germany; 2 Clinic for Anesthesiology, Critical Care Medicine and Pain Therapy, St. Georg Hospital Leipzig, Germany; 3 Clinic for Orthopedic and Reconstructive Surgery, St. Georg Hospital Leipzig, Germany; 4 Clinic for Anesthesiology and Critical Care Medicine, Martin-Luther-University of Halle-Wittenberg, Halle/Saale, Germany

**Keywords:** pulmonary hypertension, perioperative management, intraoperative monitoring, local anesthesia, general anesthesia

## Abstract

**Introduction:** It is known that pulmonary hypertension is associated with worse outcome in both cardiac and non-cardiac surgery. The aims of our retrospective analysis were to evaluate the outcomes of our patients with pulmonary hypertension undergoing major orthopedic surgery and to give experience-based recommendations for the perioperative management.

**Material and methods**: From 92 patients with pulmonary hypertension undergoing different kinds of surgical procedures from 2011–2014 in a tertiary academic hospital we evaluated 16 patients with major orthopedic surgery for perioperative morbidity and mortality.

**Results:** Regarding the in-hospital morbidity and mortality, one patient died postoperatively due to pulmonary infection and right heart failure (6.25%) and 6 patients suffered significant postoperative complications (37.5%; bleeding = 1, infection = 1, wound healing deficits = 3; dysrhythmia = 1).

**Conclusion:** Our data show that major orthopedic surgery is feasible with satisfactory outcome even in cases of severe pulmonary hypertension by an individualized, disease-adapted interdisciplinary treatment concept.

## Zusammenfassung

**Einleitung:** Patienten mit pulmonaler Hypertonie sind sowohl bei kardiochirurgischen, wie auch nicht-kardiochirurgischen Operationen einem erhöhten Risiko für schwere intra- und postoperative Komplikationen ausgesetzt. Mit unserer retrospektiven Analyse möchten wir einerseits die Ergebnisse von Patienten mit pulmonaler Hypertonie zeigen, die sich einem größeren orthopädischen Eingriff unterziehen mussten und andererseits auf unseren Erfahrungen basierende Empfehlungen zum perioperativen Management dieser Patienten geben.

**Material und Methoden:** Im Zeitraum von 2011–2014 wurden insgesamt 92 Patienten mit schwerer pulmonaler Hypertonie in verschiedenen chirurgischen Disziplinen unserer Einrichtung operiert; von diesen untersuchten wir 16 Patienten mit größeren orthopädischen Eingriffen hinsichtlich der perioperativen Komplikationsraten und Sterblichkeit.

**Ergebnisse:** Ein einziger Patient verstarb im perioperativen Verlauf an einer Pneumonie mit konsekutivem Rechtsherzversagen (6,25%); bei 6 Patienten kam es zu gravierenden Komplikationen (37,5%; Blutung = 1, Infektion = 1, Wundheilungsstörungen = 3; Herzrhythmusstörungen = 1).

**Schlussfolgerungen: **Unsere Ergebnisse zeigen, dass auch größere orthopädische Operationen bei Patienten mit schwerer pulmonaler Hypertonie mit einem höheren, aber aus unserer Sicht vertretbaren perioperativen Risiko durchführbar sind und empfehlen dafür ein individualisiertes, interdisziplinäres Behandlungskonzept.

## Introduction

Patients with severe pulmonary hypertension (PH) are at increased risk for perioperative morbidity and mortality when undergoing all kinds of cardiac and non-cardiac surgery. Stress, pain, intra-/postoperative mechanical ventilation, and trauma-induced inflammation can further increase pressure and resistance within the pulmonary arteries and cause right heart failure. Available data show a perioperative mortality of 7–24% – depending on the primary disease and the type of surgical intervention – with the highest risk for pregnant women and patients undergoing emergency interventions [1], [2], [3], [4], [5], [6]. Conditions that cause one or more perioperative complications in 42% of all patients were heart failure of NYHA class II or higher, a history of pulmonary embolism, high-risk surgery (e.g., thoracic or major abdominal surgery), and an anesthesia duration of more than 3 hours [1]. Risc factors for major complications in these patients were an elevated right atrial pressure, a six minute walking distance <399 m, the need for emergency surgery and the perioperative use of vasopressors, shown by Meyer et coworkers in a large international prospective survey [6]. Even postoperatively PH patients have significantly increased risk for hemodynamic instability, heart failure, post-operative sepsis, and respiratory failure; and required significantly prolonged postoperative ventilation and a longer intensive-care stay [7]. 

The perioperative management of patients with major pulmonary hypertension presents a great challenge to both anesthesiologists and surgeons. Little is known about the special management and outcome of orthopedic patients with severe pulmonary hypertension, therefore the aim of our paper is to present our clinical experiences with these special surgical patients and to formulate experience-based recommendations for the perioperative management. An interdisciplinary team with anesthesiologists, surgeons, pulmologists, and cardiologists will play a central role in achieving the best outcome for this high-risk population. 

## Definition, diagnosis, classification and treatment of pulmonary hypertension

Pulmonary hypertension (PH) represents a hemodynamic condition which occurs either idiopathic, hereditary or as a consequence of other diseases (updated classification; Table 1 (Tab. 1)) [8]. Based on data of a large retrospective analysis it has been found that the mean pulmonary artery pressure in healthy persons is 14 ±3 mmHg; PH is defined as PAPm ≥25 mmHg at rest [8], [9]. Therefore it is necessary to measure the mean pulmonary artery pressure (PAPm) during right-heart catheterization to diagnose PH. Invasive measurement of the pulmonary pressure includes the ascertainment of the pulmonary artery wedge pressure (PAWP) in order to distinct between precapillary pulmonary hypertension (PAWP ≤15 mmHg) and postcapillary pulmonary hypertension (due to left heart disease; PAWP >15 mmHg; Table 2 (Tab. 2)) [10], [11]. 

Pulmonary hypertension as a disease of pulmonary arteries (Group 1 = pulmonary arterial hypertension (PAH); Group 4 = chronic thromboembolic pulmonary hypertension (CTEPH); Table 1 (Tab. 1)) have a poor prognosis. Moreover, whenever PH occurs in the course of a disease (e.g. Group 2 = left heart disease; Group 3 = lung disease; Table 1 (Tab. 1) and Table 2 (Tab. 2)), the prognosis of this disease worsens [10], [11]. In view of prognosis and different therapeutic approaches the diagnostic algorithm comprises numerous examinations and has two aims: diagnosis and determining the type of pulmonary hypertension.

The treatment of choice for pulmonary hypertension due to left heart disease (Group 2), due to lung disease (Group 3) or due to unclear multifactorial mechanisms (Group 5) is the state-of-the-art therapy of the underlying disease. Patients with CTEPH should be evaluated for thromboendarterctomy. If a patient with CTEPH is not eliglible for thromboendarterectomy a treatment with the guanylate cyclase stimulator riociguat is a therapeutic option [12]. The decision on how to treat CTEPH patients should be made at an expert center based upon interdisciplinary discussion among internists, radiologists, and expert surgeons. Regardless of therapeutic approach patient with CTEPH should receive life-long anticoagulation, usually with vitamin K antagonists [12].

In recent years, various new pulmonary vasodilators have been successively tested for their effects in clinical trials and launched on the market. However, these drugs are only approved for the therapy of PAH (Group 1, Table 1 (Tab. 1)). They exert their effects using different signaling pathways, i.e. the endothelin signaling pathway (endothelin receptor antagonists: bosenthan, ambrisentan, macitentan), the prostacyclin signaling pathway (prostacyclin analogues: iloprost, epoprostenol, treprostinil), and the NO signaling pathway (phosphodiesterase-5 inhibitors: sildenafil, tadalafil; guanylate cyclase stimulator: riociguat). Table 3 (Tab. 3) gives an overview of all approved substances [13], [14], [15]. Patients with an inadequate response to monotherapy should be treated with a combination of two compounds. The updated treatment algorithm suggests, that patients with PAH and WHO functional class II or III benefit from an initital combination therapy [16]. In case of failure of medical therapy patients should be considered for lung transplantation.

## Patients

Due to close collaboration with several lung centers patients with pulmonary hypertension of all origins are common in our anesthesiological patient population. From 92 patients with severe PH (definition of “severe”: verification of elevated mean pulmonary arterial pressure by right heart catheter, history or presence of functional class III, need for specific drug therapy), who underwent major surgical procedures at St. Georg Hospital Leipzig between 2011 and 2014, we selected 16 patients with major orthopedic interventions. All listed patients suffered from precapillary forms of PH. We retrospectively evaluated the patients cohort for in-hospital mortality and major complications as bleeding, all kinds of infections, cardiac and neurologic complications, and wound healing deficits. 

## Methods

### Interdisciplinary preoperative evaluation and diagnostics

Pulmonary hypertension affects several organ systems simultaneously (lung, heart, vascular system), therefore preoperative evaluation should be considered as a joint task of anesthesia, surgery, pulmonology, and cardiology [17]. The purpose of these preparations should be, on the one hand, to evaluate the functional state of the heart and lung organ systems as good as possible. On the other hand the patient’s initial conditions should be optimized as far as possible by adjusting the current specific medication and treatment of comorbidities. Both approaches are appropriate to minimize the individual risk of complications. The diagnostics listed below are not obligatory for all patients, but should be composed individually dependent from basic disease and functional state.

**Figure 1:**
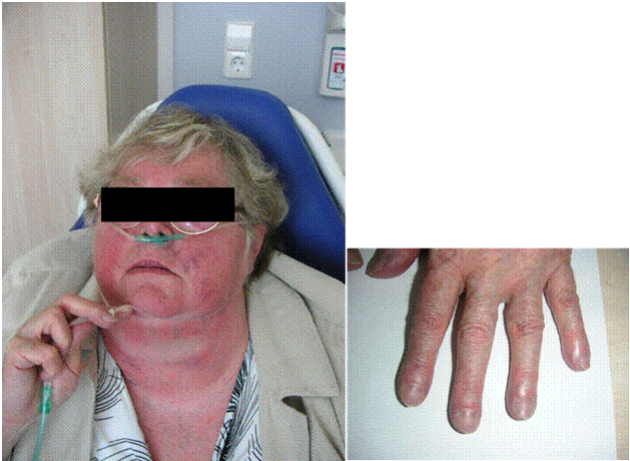
Clinical findings in a patient with chronic right heart insufficiency and severe pulmonary hypertension

*Clinical examination:* The clinical symptoms of pulmonary hypertension are largely unspecific, often overlooked or misinterpreted in early stages of the disease (Table 4 (Tab. 4)) [18]. The most common but, unfortunately, very unspecific symptom is stress-induced dyspnea. In addition to obtaining a detailed medical history, the clinical investigation should focus on symptoms for right-sided heart failure. In late-stage diseases in particular, obstruction of the jugular veins, peripheral edema, hepatomegaly, hepatojugular reflux, and ascites are probable (Figure 1 (Fig. 1)). The functional classification of pulmonary hypertension is similar to the criteria of NYHA/WHO (Table 5 (Tab. 5)) [19]. 

*Thoracic X-ray:* Characteristic findings for pulmonary hypertension include right-ventricular hypertrophy, dilation of the central pulmonary arteries, and vascular rarefaction in the peripheral pulmonary parenchyma [18]. Depending on the genesis, the specific symptoms of the primary disease (e.g., chronic hypoxia for pulmonary diseases) may also be diagnosed. The longer pulmonary hypertension persists, the more visible the changes become on the thoracic X-ray image. 

*ECG:* Characteristic changes are also more pronounced if major right-ventricular load already prevails [18]. Typical symptoms for pulmonary hypertension are right bundle branch blocks and “snow-shovel”-shaped ST segment depressions in the precordial leads. 

*Pulmonary function examination:* It is recommended, in particular for patients with chronic lung diseases, that an examination of pulmonary function and an arterial blood gas analysis is conducted prior to the surgical intervention [20]. 

*Spiroergometry:* Important information for estimating the severity and progression of the disease can be obtained using spiroergometry [18]. The most important parameters are the maximum oxygen uptake (peak VO_2_), the ventilatory equivalent ratio for CO_2_ (VE/VCO_2_), and the partial pressure of end-tidal carbon dioxide (PETCO_2_). 

*Echocardiography:* Echocardiography is currently the non-invasive method with the highest sensitivity and specificity for diagnosing PH. Hypertrophic and dilated right ventricle, a flattened ventricular septum (possibly with paradoxical motion), a dilated right atrium, and a dilated inferior vena cava are typical signs of pulmonary hypertension [21]. Independent prognostic factors are an enlarged right atrium (RA surface >27 mm²), the presence of pericardial effusion, and impaired global pumping capacity of the right ventricle [21]. 

*Right heart catheterization:* For patients with late-stage pulmonary hypertension, current hemodynamic data (not older than 3–4 months) should be available at the time of the surgical intervention. The findings of right heart catheterization provide important leads for evaluating the range of hemodynamic parameters in the perioperative course and for determining the point at which therapeutic measures should be initiated [22]. 

*Optimization of primary-disease therapy:* Before surgical intervention, medication should be critically examined from a pulmonological and cardiological perspective with a view to possible optimization. At the time of surgery, the patient should ideally have been in a stable condition for an extended period of time. 

*Risk disclosure:* Based on our knowledge of significantly increased perioperative morbidity and mortality, the critical assessment of risks and possible benefits of surgical intervention is of outstanding importance. Patients should be thoroughly informed about possible risks long before surgery in order to give them sufficient time for consideration. Close family members should also be involved if possible. Depending on the initial conditions of each patient, they may also need to be explicitly informed about the possibility of severe complications that can lead to extended hospitalization or even death. 

### Intraoperative monitoring

The intraoperative monitoring should be adapted individually to the severity of the disease and the invasiveness of the surgical procedure. To date, there is no evidence to suggest that any specific type of monitoring has an influence on patient morbidity and mortality. However, the authors believe that early recording of deviations from the initial conditions (in particular in relation to right heart function) can make a decisive contribution to recognizing and avoiding severe complications from the outset. 

Whereas basic monitoring can be considered sufficient for minor and medium procedures in functional state II, all major interventions and those in functional state III should be carried out under extended monitoring (Table 6 (Tab. 6)) [22]. For intraoperative fluid management stroke volume variability (SVV) is an appropriate method of evaluating volume responsiveness, provided that the prerequisites for its use are fulfilled (sinus rhythm, ventilation) [23]. 

For all patients in the late stages of PH and existing or history of right-sided heart failure, pulmonary artery catheterization and/or transesophageal echocardiography (TEE) are the methods of choice for adequate intraoperative monitoring. The best method for evaluating preload and contractility is certainly TEE, even if it requires the presence of specifically trained personnel. The intraoperative use of pulmonary artery catheters is subject to controversial discussions in the current literature. However, all authors point out that the insertion of a pulmonary artery catheter is associated with certain risks, which must be considered when applying this monitoring method. 

In our orthopedic patients we used the pulmonary arterial catheter in 8 of 16 cases for intraoperative monitoring, this with special respect to the consequences of unexpected and massive volume displacements, or reactions to bone cement on the pulmonary pressure and right heart. 

### Selection of the anesthetic technique

Patients with late-stage pulmonary hypertension should be treated in medical centers that fulfill all conditions for qualified treatment in terms of their structure and personnel (Table 7 (Tab. 7)). 

All standard anesthetic techniques can, in principle, be applied to patients with pulmonary hypertension [24]. Regional anesthetic techniques offer the advantage of not impairing spontaneous breathing and can be used for postoperative analgesic therapy. In general, continuous techniques should be preferred to bolus administration – especially for spinal or epidural analgesia – in order to avoid uncontrolled drops in blood pressure, which can endanger myocardial perfusion [22], [24]. In our orthopedic patients with PH, spinal and epidural catheter techniques are also preferred. By fractionated administration, the required dose can be delivered without any significant effects on hemodynamics. Plexus or nerve catheters (sciatic or femoral nerve) are recommended for surgical procedures involving the extremities in particular, as they do not affect hemodynamics, have low failure rates, and ensure treatment of postoperative pain. Nearly all patients with pulmonary hypertension receive continuous anticoagulant therapy, this fact must be taken under consideration when planning the intervention and the regional anesthetic procedures. Recommendations in relation to this issue are provided in the current guidelines [25]. 

Particularly in the later stages of pulmonary hypertension or in diseases affecting the lung, patients cannot be subjected to remaining in a flat position over a long period of time. In these cases, regional anesthesia should be combined with careful general anesthesia to ensure adequate oxygenation [22]. 

**Figure 2:**
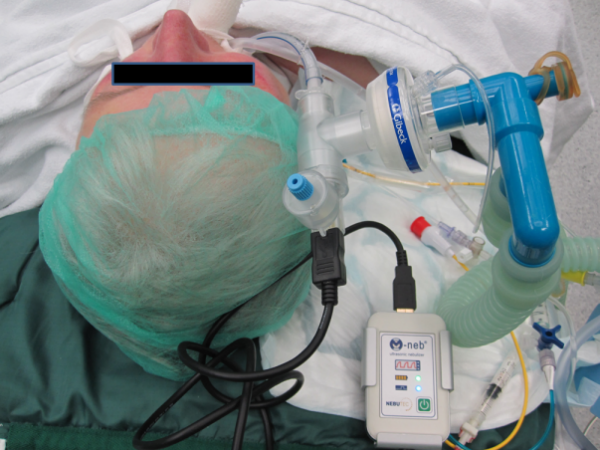
Intraoperative selective pulmonary vasodilation with inhaled iloprost via ultrasonic nebulizer (m-neb^®^, nebutec Elsenfeld, Germany) in the ventilatory circuit

In view to general anesthesia the main advantages are safe oxygenation and uncomplicated airway management, and intraoperative selective pulmonary vasodilation can – if necessary – easily be administered through the breathing circuit (Figure 2 (Fig. 2)). Anesthesia-induced systemic vasodilation and mechanical ventilation can lead to a significant drop in mean arterial pressure, which has the potential to endanger myocardial perfusion and critically affect right-ventricular contractility [26]. All standard induction anesthetics can, in principle, be used in combination with opioids, as they have no influence on pulmonary vascular resistance and oxygenation [27]. Histamine-releasing relaxants (atracurium, mivacurium) should be avoided for patients with pulmonary hypertension, as they may further increase pulmonary resistance [27]. Volatile anesthetic agents of concentrations up to 1 MAC can be administered without any negative effects on pulmonary pressure and resistance [26]. We suggest a balanced technique, mixing higher doses of opioids and low-dose volatile anesthetic agents [22]. 

In our special orthopedic patient population we – whenever possible – used a combined anesthesia technique with volatile anesthetics and regional or neuroaxial anesthesia to minimize the side effects of anesthetics and to ensure an adequate postoperative analgesia with minimal use of opioids.

### Intraoperative treatment of increased pulmonary arterial pressure

**Figure 3:**
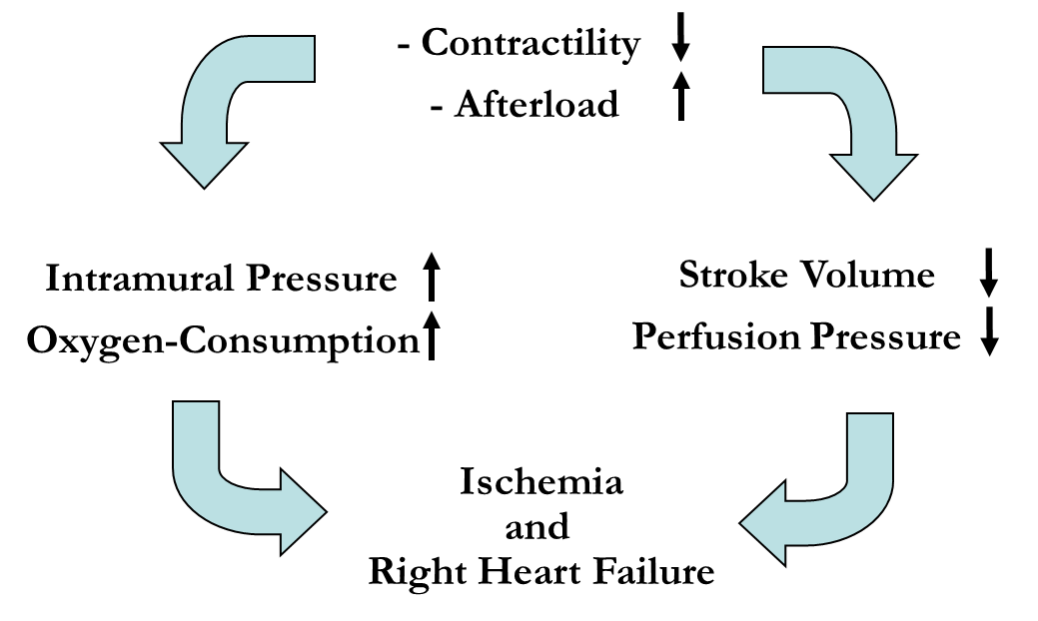
Mechanisms of acute right heart failure (adapted from [22])

The most important requirement for intraoperative management and maintenance of anesthesia is to avoid anything that could increase right-ventricular afterload or decrease contractility of the right ventricle, as both factors will ultimately lead to ischemia and right-sided heart failure (Figure 3 (Fig. 3)). 

Hypoxia is one of the strongest inducers of pulmonary vasoconstriction, therefore high inspiratory oxygen concentration should be used (FiO_2_ 0.6–1.0) to minimize the risk of hypoxic phases. Carefully performed recruitment maneuvers are able to prevent inadequate ventilation-perfusion ratios [28]. It is not clear if an intraoperative low-tidal-volume ventilation offers any benefits over “conventional” pressure-controlled ventilation; the authors would recommend to set peak pressures as low as possible (6–8 ml/kg ideal body weight) to avoid alveolar over-inflation [22]. In addition to hypoxia, acidosis and hypercapnia may also aggravate existing hypertension. Therefore, moderate hyperventilation (target PaCO_2_ of 30–35 mmHg) should be carried out under continuous blood gas analysis, but without allowing the pH value to fall below 7.4 [24], [26]. Hypothermia and shivering can considerably increase pulmonary pressure and should therefore be strictly avoided (Table 8 (Tab. 8)). 

Perioperative fluid management should be carried out rather restrictively and in a targeted manner, with adequate hemodynamic monitoring to optimize right-ventricular preload. It is very difficult to indicate general target values for these therapy forms that take account of the individual needs of this particular patient population. The target values for right-sided heart failure – e.g., after heart transplantation – certainly cannot be applied to patients with chronic pulmonary hypertension [29]. One possibility, which is favored by the authors, would be to consider the initial values measured during preoperative evaluation (right heart catheter!) as target values and to initiate specific treatment in the event of deviations ±15–20%. Blaise et al. also recommend carrying out intraoperative management in a way that allows mean pulmonary artery pressure to fluctuate in a range of 15% above or below the initial value [27]. However, clinical trials have not yet collected sufficient data to substantiate this “target corridor”. 

In general, it should be considered that patients with PH have low arterial pressure as a result of their disease and the specific therapy, and that the possibilities for compensation are very limited due to right-sided heart failure. Therefore, if mean arterial pressure falls below the critical value of 50–55 mmHg, low doses of a vasoconstrictor (e.g., noradrenalin 2–5 µg) should be administered carefully [22], [27]. 

#### Intraoperative vasodilator therapy

If an elevation in pulmonary artery pressure cannot be controlled by the symptomatic measures described above, specific medication should be induced immediately to reduce right-ventricular afterload and thus the risk of right-sided heart failure. The required vasodilators can be administered both intravenously and by inhalation. 

Administration of nitroglycerin, sodium nitroprusside, milrinone, dobutamine, or prostacyclin is recommended for intravenous vasodilation (for dosage see Table 9 (Tab. 9)) [22], [24], [27]. As the effect of these substances is not limited to the pulmonary circulation and is accompanied by systemic vasodilation, a considerable decrease in systemic mean arterial pressure may involve the risk of right-ventricular perfusion pressure falling below a critical limit [30]. In the case of hypotonic blood circulation selective pulmonary vasodilator therapy by inhalation offers several advantages over intravenous vasodilation. As alveoli and pulmonary capillaries are located in close proximity, the effect of inhaled vasodilators is direct, immediate and – in case of short-acting drugs – limited to the pulmonary vascular bed; therefore avoiding systemic vasodilation and hypotension. In addition, substances that are administered by inhalation only have an effect on ventilated lung areas, and the consecutive vasodilation in the ventilated areas therefore leads to a decrease of the pulmonary shunt and improved oxygenation [31]. 

Several substances are currently available for vasodilator therapy by inhalation in patients with pulmonary hypertension (Table 9 (Tab. 9)). Nitric oxide (NO) was the first substance to be used in inhalation therapy for diseases accompanied by a pathological elevation of pulmonary pressures [32]. Although it can be administered using a tight-fitting breathing mask, this is difficult to apply in clinical practice. Patients only tolerate the maneuver for a very short period of time, the dosage is difficult to control and high personnel capacity is required. The short-acting prostacyclin triggers vasodilation by elevating cAMP in the vascular muscle cells. Its half-life is only 2–3 minutes, and so the inhalation of prostacyclin also demands controlled ventilation [33]. The effectiveness of prostacyclin appears to be comparable with that of NO [33]. 

Due to its longer half-life of 20–30 minutes, the stable prostacyclin analog iloprost can be administered intermittently to ventilated and spontaneously breathing patients, and therefore offers significant advantages over short-acting inhaled vasodilators [34]. It is approved for the treatment of pulmonary arterial hypertension. In addition, many articles have been published on off-label use in patients following cardiac surgical interventions, as well as those with chronic thromboembolic pulmonary hypertension and severe right-sided heart failure [35], [36]. Iloprost should be administered using an ultrasonic nebulizer to ensure that particles of a specific size (3–5 µm) are inhaled and actually reach the alveoli. In relation to the inhalation of iloprost, it should be noted that, due to its longer half-life, systemic effects cannot be completely excluded when administering higher doses. 

Another option for vasodilator therapy by inhalation, which, to date, has not been thoroughly described, is the inhalation of milrinone [37]. Dosage indications vary between 2 mg when testing pulmonary vascular responsiveness in cardiac transplantation candidates and 5 mg for application after cardiac surgical procedures [38]. Although no side-effects have been described in the articles published to date, the strong acid solution could potentially cause airway irritations and should therefore be diluted before administration [38]. Even so, combining a phosphodiesterase inhibitor with iloprost may still be worthy of consideration, and may prove effective in patients who show little or no response to the administration of iloprost alone, or who have an acute and dramatic elevation of pulmonary artery pressures [39]. All inhalable substances should not be administered to patients with decompensated left-sided heart failure because, in the event of massive pulmonary back pressure caused by left-ventricular afterload reduction, the selective pulmonary vasodilation may trigger an acute pulmonary edema [22]. 

### Postoperative recovery and analgesic therapy

Patients with pulmonary hypertension are at risk of developing elevated pulmonary pressure and right-sided heart failure not only during the perioperative phase itself, but also in the postoperative course. These patients should therefore be placed under intense postoperative monitoring for a period appropriate to the degree of surgical trauma; the target monitoring time should be between 24 hours for small interventions and several days for major procedures (abdominal and thoracic surgery, major urological interventions). Depending on the patient’s initial condition (functional classification), hemodynamic monitoring may need to be continued postoperatively until pulmonary pressures and right-sided heart functions have stabilized at the preoperative level. For our orthopedic patients, the mean monitoring time in intensive or intermediate care was 32.4 hours.

In this phase, sufficient analgesic therapy can make a decisive contribution to the avoidance of pulmonary complications. In the ideal case, analgesic therapy in the form of continuous regional anesthesia can be organized in a way that avoids higher doses of opioid-based analgesics. The basic treatment of patients with pulmonary hypertension therefore includes daily visits by pain management nurses. The specific therapy for pulmonary hypertension should be resumed at the preoperative dosage as soon as possible. In the postoperative course, it is also advisable to treat pressure elevations with iloprost inhalation, which can also be administered intermittently due to its long half-life. 

## Results

The clinical data, operation and anesthesia characteristics and outcome data are shown in Table 10 (Tab. 10) and Table 11 (Tab. 11). All patients suffered from precapillary forms of pulmonary hypertension: PH-group I in 8 patients, group III in 5 patients, and group IV in 3 patients. At time of surgery, all patients were on specific medical treatment for PH: 10 patients with single-drug therapy and 4 patients with combination therapy. All patients were actually at functional class III or higher or had at least one history of class III. The meanPAP-values in this group of patients was 39.3 mmHg ±6.1 mmHg.

Only in 6 patients a “pure” general anesthesia (GA) was choosen for surgery, in 6 patients GA was combined with regional techniques, and in 4 patients the intervention were made exclusively under regional anesthesia. 

Regarding the in-hospital morbidity and mortality, one patient died postoperatively due to pulmonary infection and right heart failure (6.25%) and 6 patients suffered significant postoperative complications (37.5%; bleeding = 1, infection = 1, wound healing deficits = 3; dysrhythmia = 1).

## Discussion

Pulmonary hypertension is a major reason for elevated perioperative morbidity and mortality, even in non-cardiac surgical procedures. With our data we can show, that perioperative complications and outcome of patients in major orthopedic surgery are comparable to the data known from other surgical disciplines. The group of our patients is too small to discuss seriously about the mortality, but the rate of postoperative complications is similar to the data of Ramakrishna and Rodriguez [1], [2]. Price et al. reported about 28 PH patients having non-cardiac and non-obstetric surgery under general or regional anesthesia: at the time of surgery, 75% of patients were in NYHA functional class 1–2. Deaths occurred in 7% of patients and peri-operative PH-related complications occurred in 29% of patients [40]. In a smaller case series of 21 patients with moderate to severe PH, Minai et al. showed an 18% mortality rate [41]. Considering that our orthopedic patients were throughout at functional class III, our results show that careful preoperative evaluation and optimization may contribute to minimize the perioperative risk. In the retrospective study of Kaw the mortality rate was very low with 1% [7]. But in this study population, there were a great number of patients with pulmonary hypertension due to left heart failure (Group 2). The anesthetic management of patients with left heart desease differ significantly from those with precapillary forms of pulmonary hypertension, therefore the results are not comparable.

It is consensus in all reviews that patients with pulmonary hypertension should be thoroughly prepared for the intervention by an interdisciplinary team. Pilkington and coworkers support our concept, that the pre-operative evaluation of a patient with established pulmonary hypertension should be based on a risk assessment that takes into account their functional state, severity of the disease and type of surgery proposed [24]. Established PAH therapies should be continued in the peri-operative period and when oral formulations cannot be used, temporary administration of inhaled (NO, nebulised prostacyclin) or intravenous (prostacyclin, sildenafil) therapy should be considered [24].

Various anesthetic techniques have been used in patients with pulmonary hypertension. Neuroaxial regional anesthesia was previously thought harmful in patients with PH because of the hemodynamic compromise following sympathetic blockade; however, as shown in our orthopedic patients, using a spinal or epidural catheter technique with low intrathecal dose or careful incremental epidural top-ups minimizes this potential drop in afterload. Regional techniques have also been used in patients with PH in general surgery [1], [42]. On the other hand, regional anesthesia may be inappropriate for many surgical procedures, as well as in emergency cases. Based on our experiences we recommend regional anesthesia techniques, alone or – if necessary – in combination with moderate general anesthesia as method of choice for patients with PH undergoing orthopedic surgery.

The intraoperative management should focus on maintaining right ventricular cardiac output, avoiding systemic hypotension, and avoiding all circumstances that could contribute to exacerbating pulmonary hypertension (hypoxemia, hypercapnia, acidosis, hypothermia, hypervolemia). In this context, the intraoperative management should also pay attention to the fluid management: the balance between adequat right-ventricular filling and hyperhydration is often difficult to find and requires a extended intraoperative hemodynamic monitoring and the knowledge of preoperative hemodynamics. We prefer a restricted fluid substitution, adjusted to the individual needs and intraoperative hemodynamics. Hypotonia should not be tolerated and treated with fractional or continuous low-dose vasoconstrictots to maintain an adequate myocardial perfusion. 

A method of treating elevations in pulmonary pressure by inhalation of short-acting pulmonary-selective vasodilators should be available intra- and postoperatively. During the postoperative phase, patients must be monitored continuously and receive sufficient analgesic therapy over an adequate period of time. 

It is important to emphasize that the selection of the anesthesia team is just as crucial as the selection of the anesthetic technique to be used. It is essential to have not only excellent anesthesiological expertise on hand, but also specific knowledge of the pathophysiology of pulmonary hypertension and right-sided heart failure, the interpretation of hemodynamic data, and the corresponding concepts of complex medical treatment.

All in all, perioperative management of patients with pulmonary hypertension presents an interdisciplinary challenge that requires the adequate involvement of anesthetists, surgeons, pulmonologists, and cardiologists alike. Our data show that orthopedic surgery is feasible with satisfactory outcome even in cases of severe pulmonary hypertension by an individualized, disease-adapted interdisciplinary treatment concept.

## Notes

### Authorship

HJS and JG equally contributed to the writing of this article.

### Conflicts of interest

HJS: none; JG: honoraria for lectures from Pulsion Medical Systems; AS: honoraria for lectures and travel reimbursements from Bayer Healthcare, Actelion, Glaxo Smith Kline, CSL Behring, Boehringer Ingelheim; SG: none; MM: none; AG: none; RG: none; EC: none.

## Figures and Tables

**Table 1:**
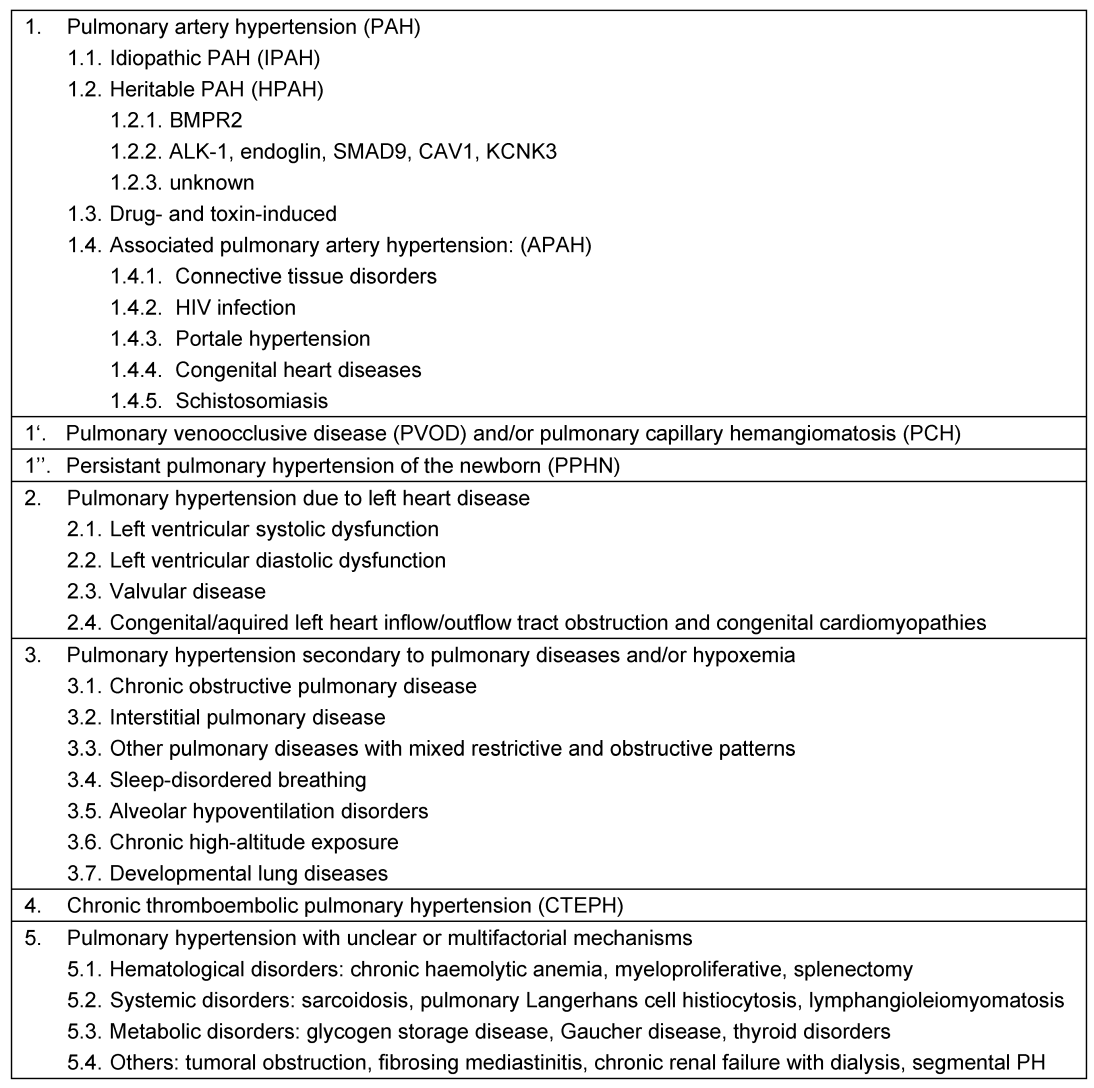
Classification of pulmonary hypertension (Nice [8])

**Table 2:**
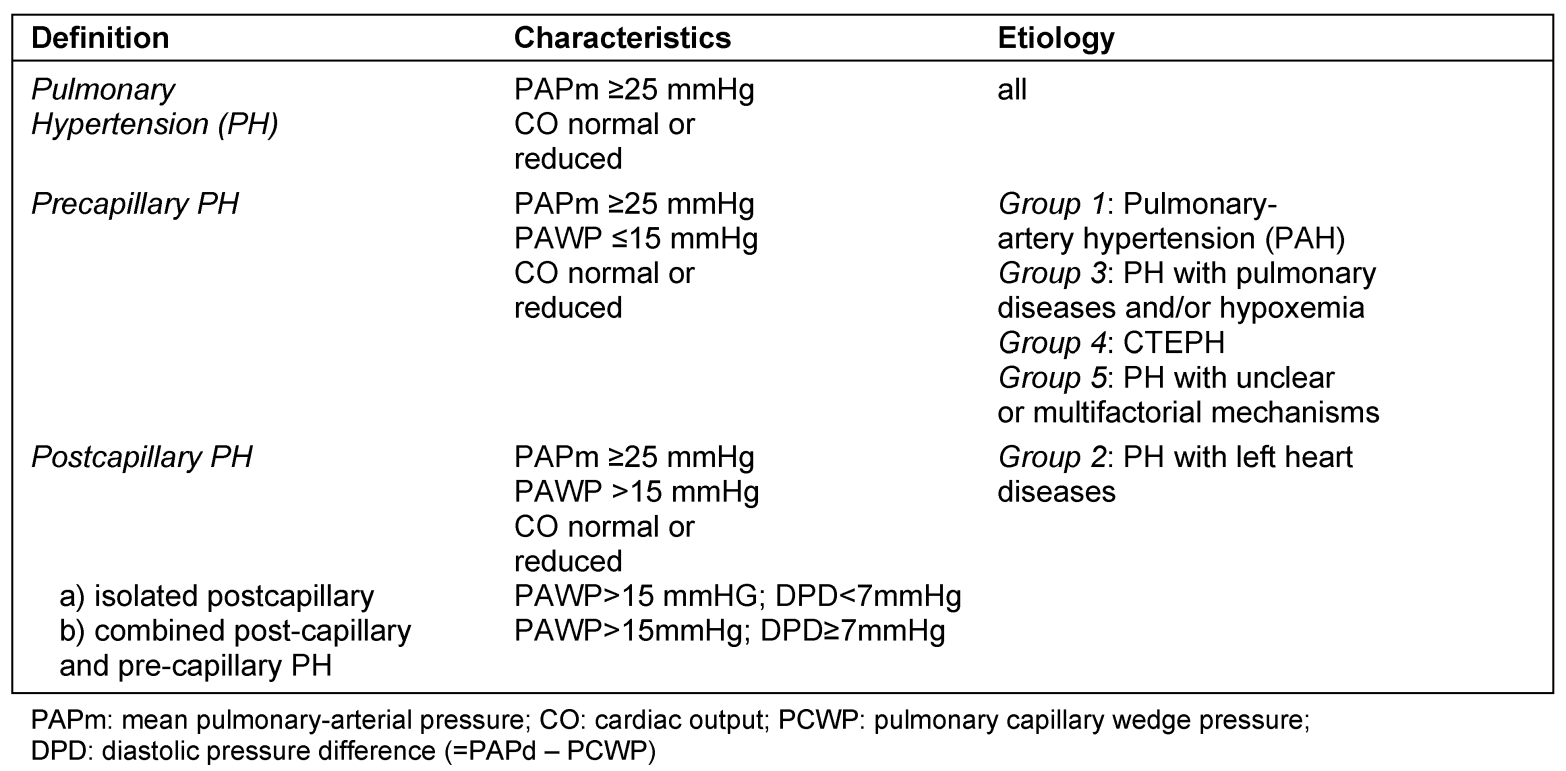
Hemodynamic characteristics in patients with pulmonary hypertension (mod. [9], [10])

**Table 3:**
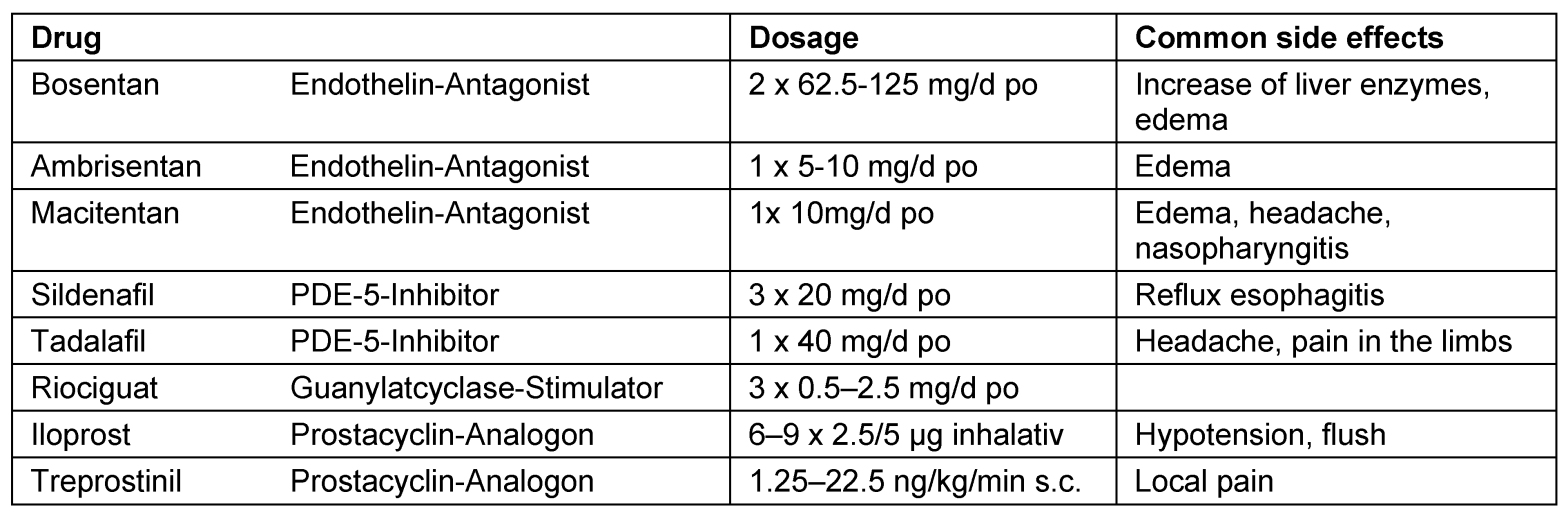
Therapy of pulmonary hypertension: approved drugs (mod. [12], [13], [14])

**Table 4:**
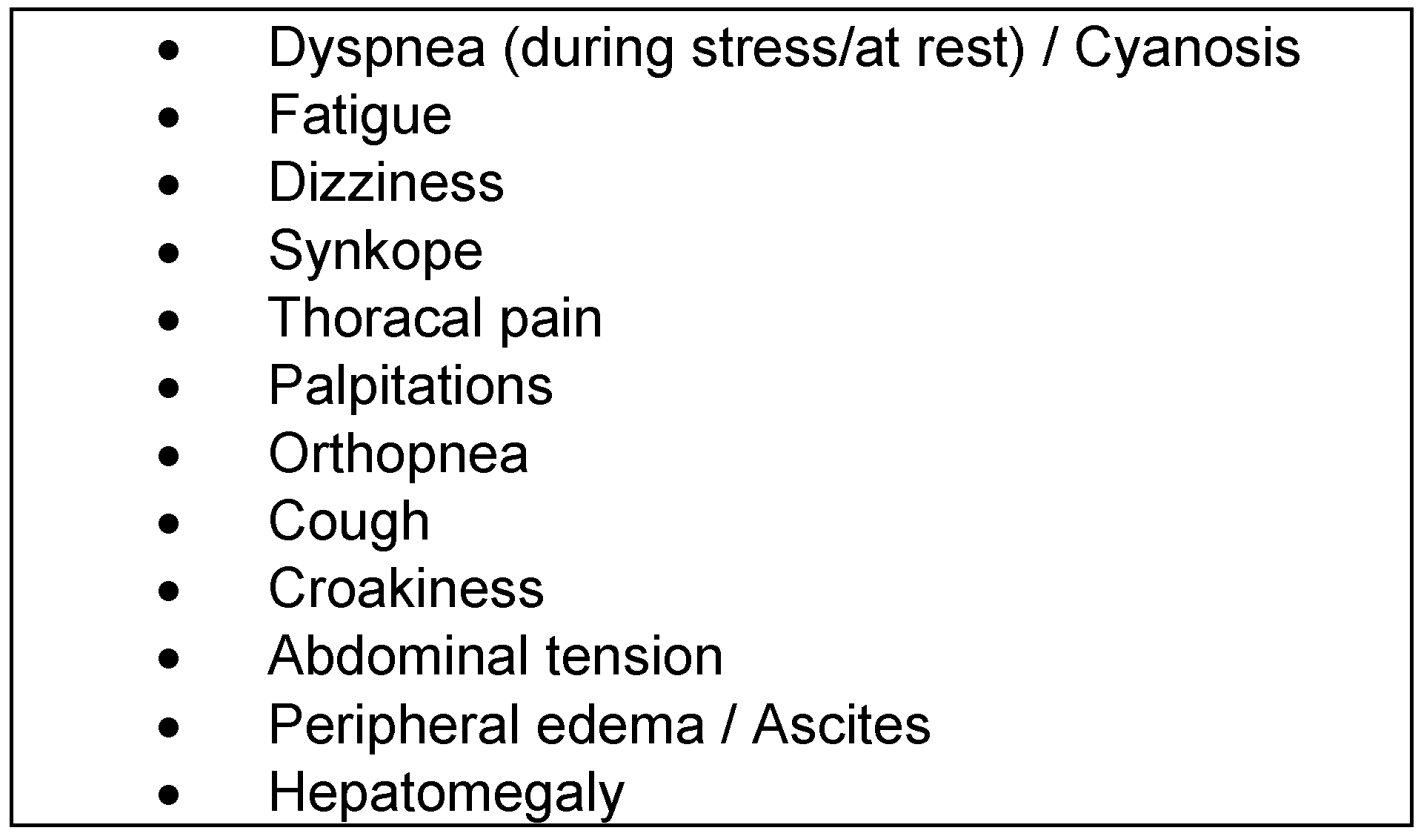
Clinical findings in patients with pulmonary hypertension (mod. [17])

**Table 5:**
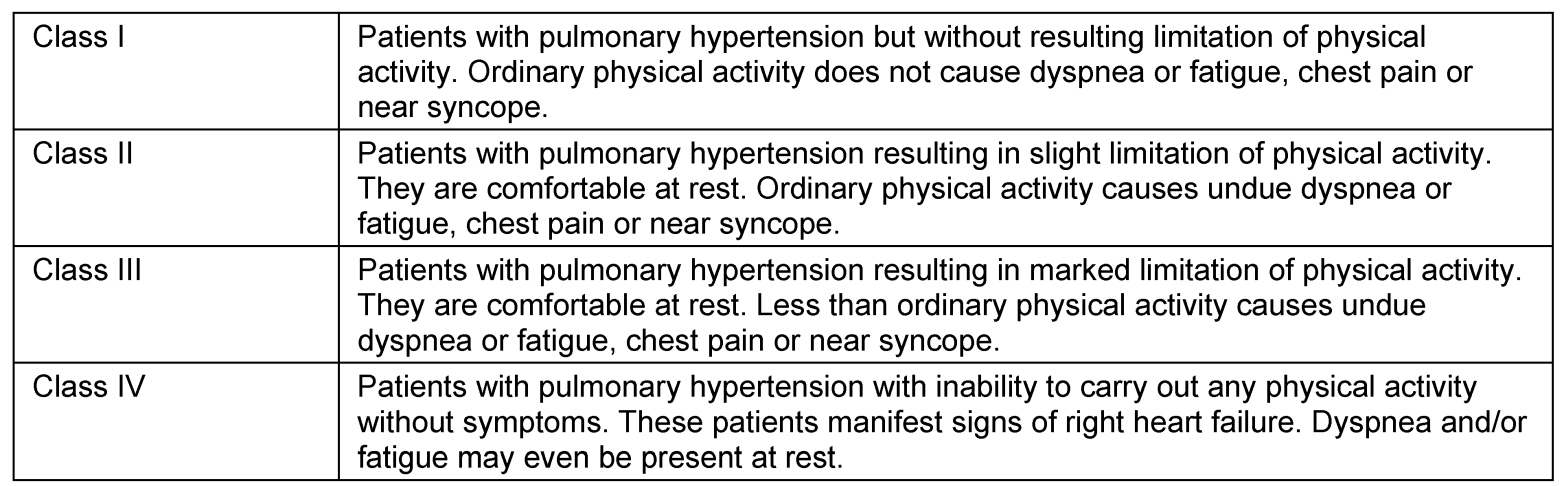
Functional classification of pulmonary hypertension (WHO 1998) [18]

**Table 6:**
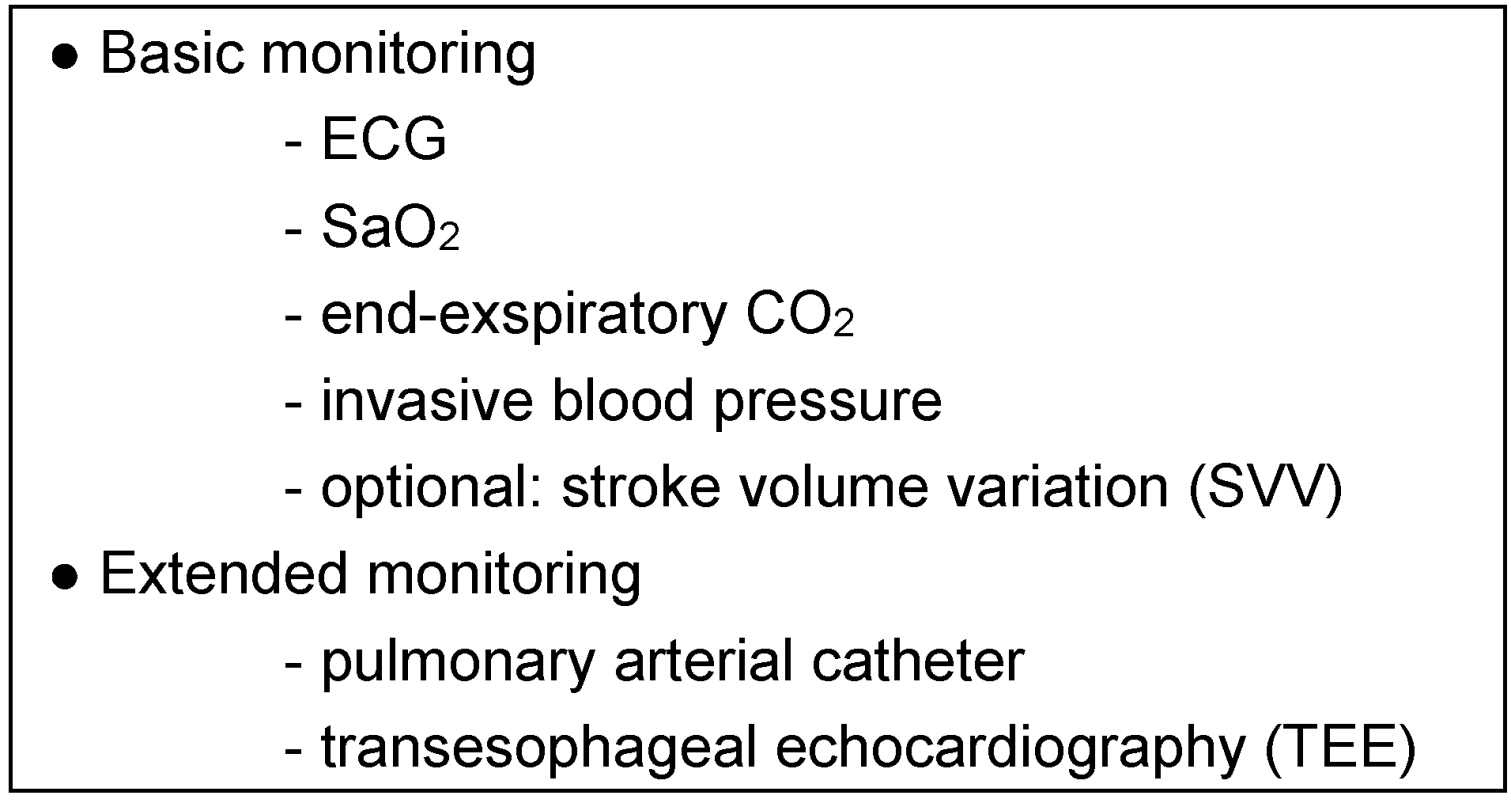
Intraoperative monitoring: recommendation for patients with PH (mod. [22])

**Table 7:**
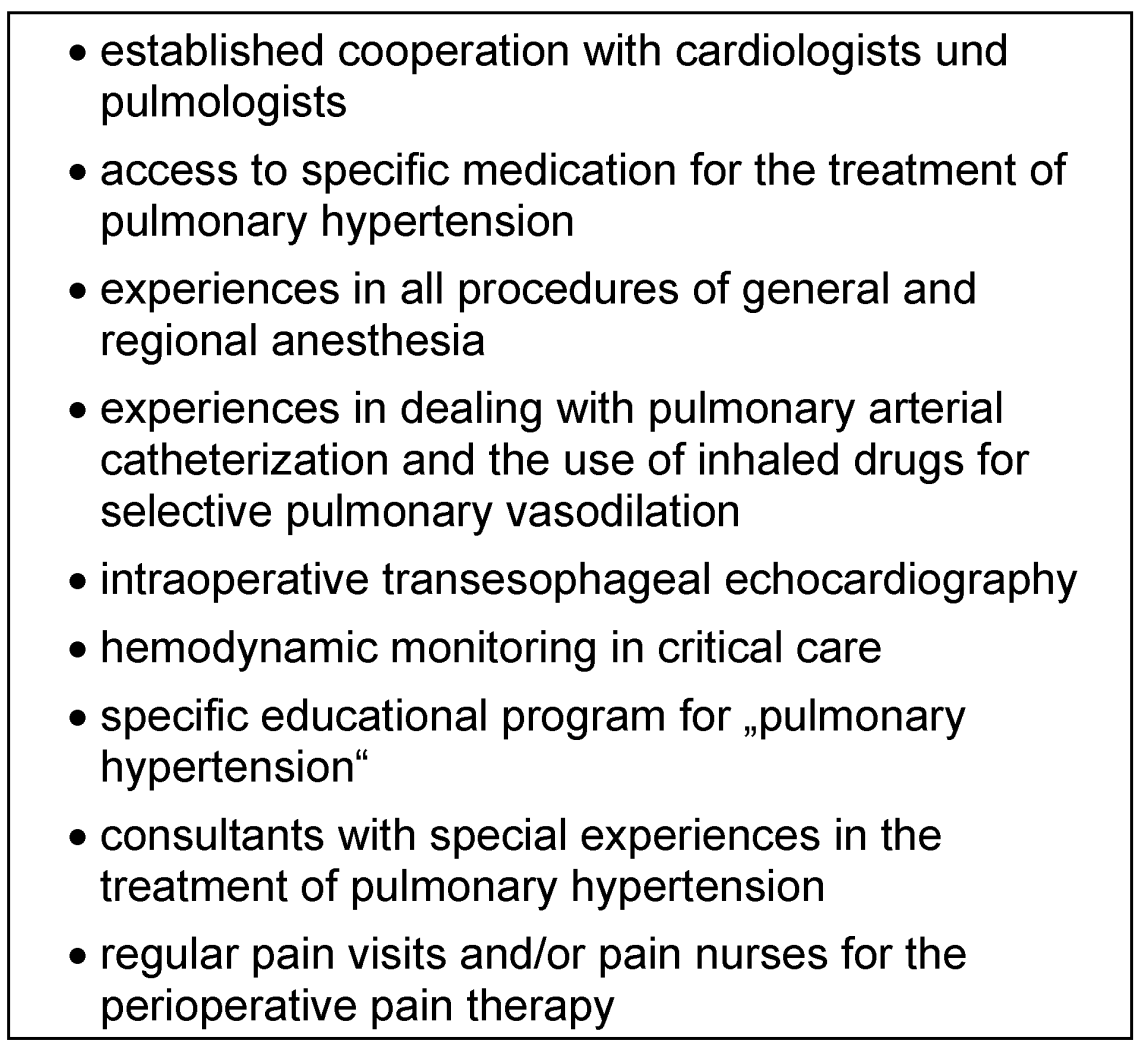
Authors recommendations: human, structural and technical requirements for the perioperative management of patients with severe pulmonary

**Table 8:**
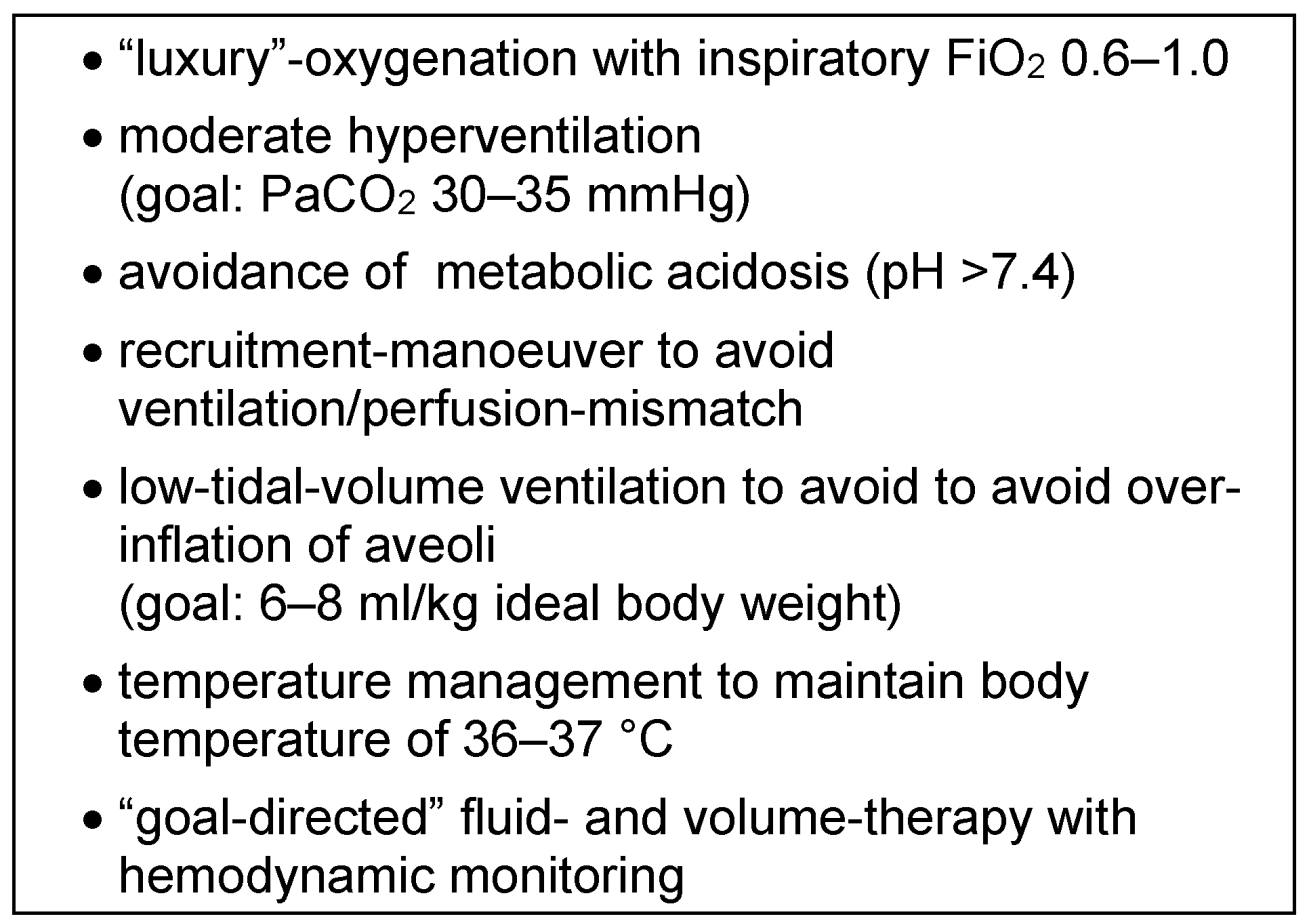
Intraoperative “basic treatment” to avoid an increase of pulmonary arterial pressure (mod. [22], [24], [26])

**Table 9:**
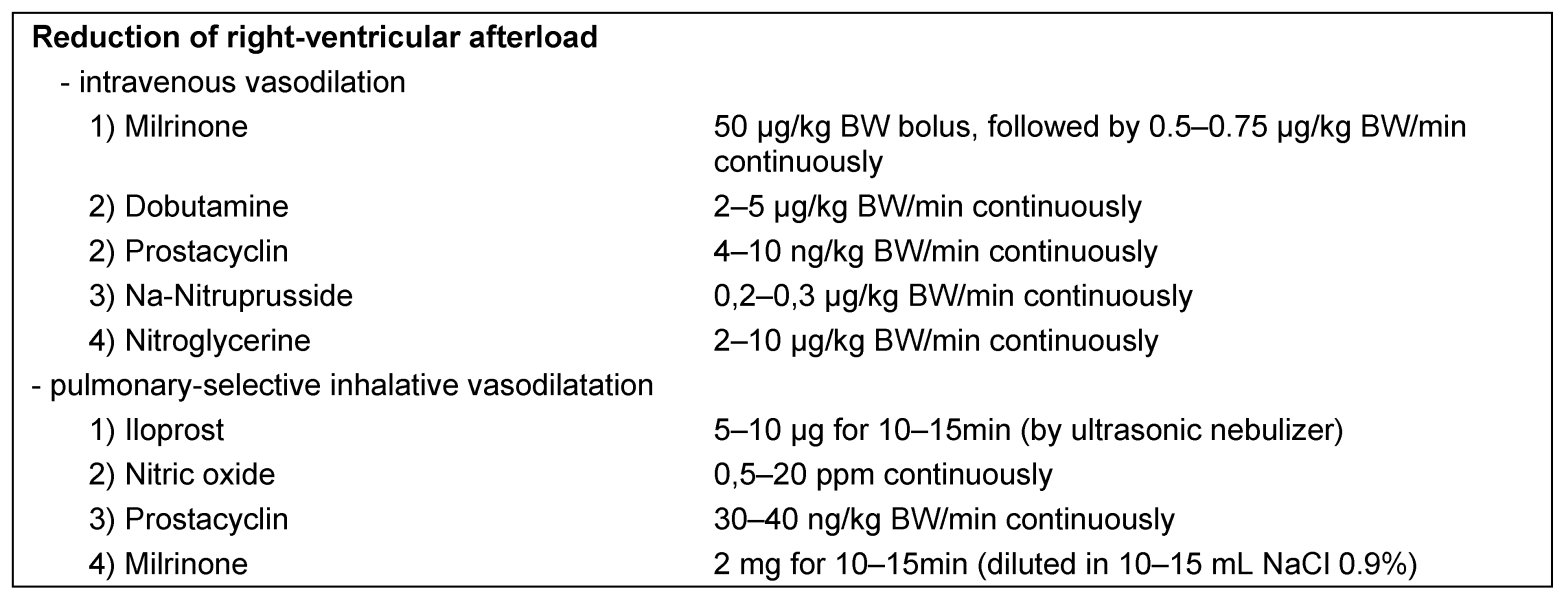
Specific interventions for therapy of intra- and/or postoperative increase of pulmonary arterial pressure (mod. [22], [24], [27])

**Table 10:**
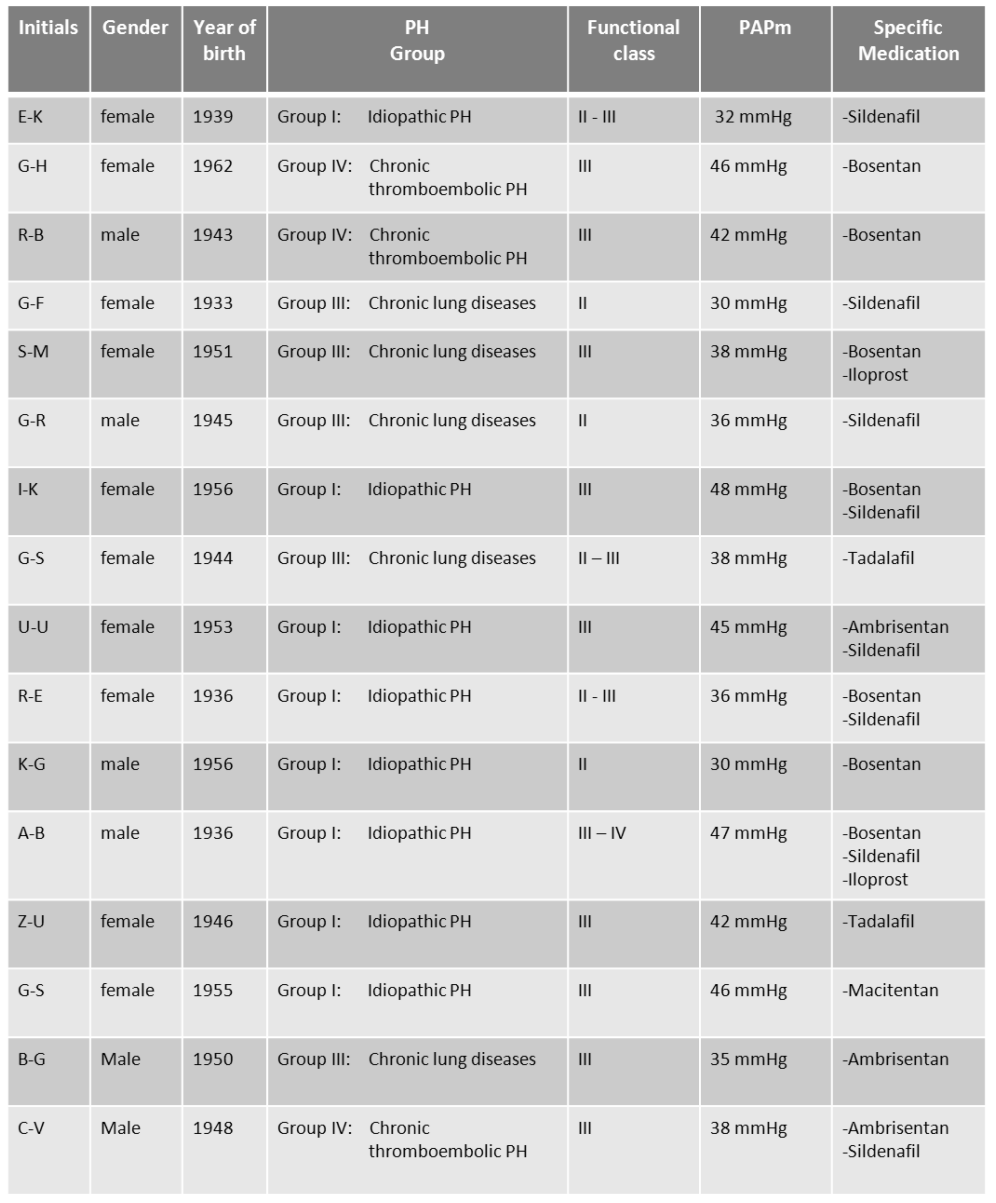
Patients characteristics

**Table 11:**
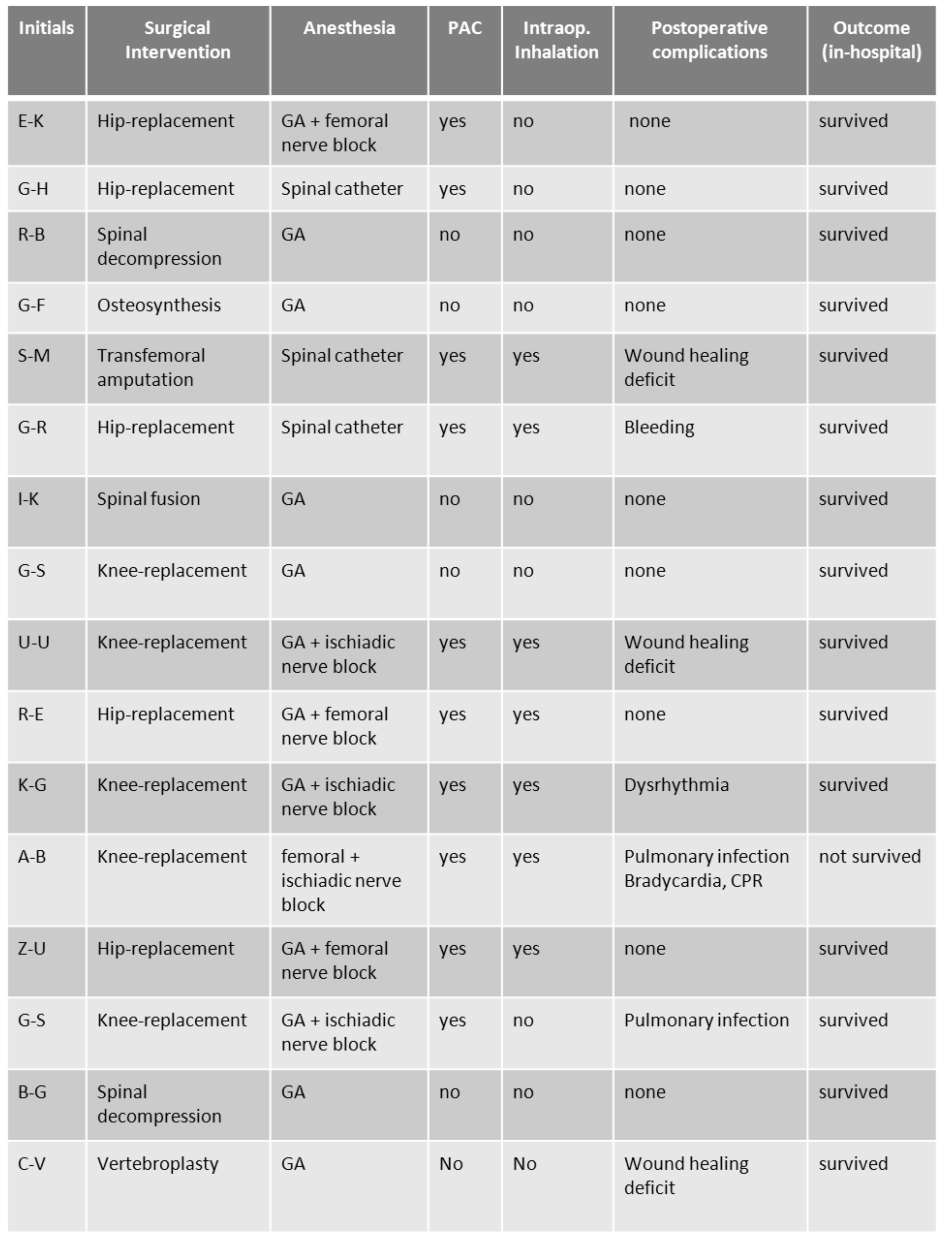
Operation characteristics, complications and outcome
